# Clozapine and mortality: A comparison with other antipsychotics in a nationwide Danish cohort study

**DOI:** 10.1111/acps.13267

**Published:** 2020-12-25

**Authors:** Yvonne van der Zalm, Leslie Foldager, Fabian Termorshuizen, Iris E. Sommer, Jimmi Nielsen, Jean‐Paul Selten

**Affiliations:** ^1^ Rivierduinen Institute for Mental Health Leiden The Netherlands; ^2^ Deptartment of Psychiatry & Neuropsychology School for Mental Health and Neuroscience Maastricht University Medical Center Maastricht The Netherlands; ^3^ Department of Animal Science Aarhus University Tjele Denmark; ^4^ Bioinformatics Research Centre Aarhus University Aarhus Denmark; ^5^ Department of Neuroscience and Department of Psychiatry University Medical Center Groningen Groningen The Netherlands; ^6^ Mental Health Centre Glostrup Mental Health Services University of Copenhagen Glostrup Denmark

**Keywords:** psychosis, clozapine, mortality, outpatient treatment

## Abstract

**Objective:**

To compare the mortality in people using clozapine to that of people using other antipsychotics.

**Methods:**

Danish incidence cohort of 22,110 patients with a first diagnosis of non‐affective psychotic disorder (1995–2013) and a prevalence cohort of 50,881 patients ever diagnosed with such a disorder (1969–2013). Hazard ratios (HR) were calculated for the antipsychotic drug used at the time of death (“current use”: incidence and prevalence cohort) and for the drug used for the longest at that moment (“cumulative use”: incidence cohort), using a Cox model with adjustment for somatic comorbidity. Clozapine was the reference drug.

**Results:**

As for current drug use, the risk of suicide was higher among users of other antipsychotics in the incidence (HR_adj_ = 1.76; 95% CI 0.72–4.32) and prevalence (HR_adj_ = 2.20; 95% CI 1.35–3.59) cohorts. There was no significant difference in all‐cause or cardiovascular mortality in the two cohorts. Cumulative use of clozapine was not associated with an increased cardiovascular mortality. Cumulative use of other antipsychotics for up to 1 year was associated with a lower all‐cause mortality and suicide risk than a similar period of clozapine use (all‐cause: HR_adj_ = 0.73; 95% CI 0.63–0.85, suicide; HR_adj_ = 0.65; 95% CI 0.46–0.91).

**Conclusion:**

The results indicate that the use of clozapine is not associated with increased cardiovascular mortality. We found opposing trends toward a lower risk of suicide during current use of clozapine and a higher risk of suicide associated with cumulative use up to 1 year. This suggests that clozapine cessation marks a period of high risk of suicide.

**Significant outcomes**

- Despite the long‐term follow‐up, we found no major differences in cardiovascular mortality between users of clozapine and users of other antipsychotics.
- The findings add to an increasing body of evidence that clozapine use is associated with a reduced risk of suicide.
- Clinicians should carefully monitor patients from whom clozapine has been withdrawn within the first year, because these patients are at an increased risk of suicide.

**Limitations**

- Information was available on dispensing of drugs, but not on actual use of drugs.
- Drug use during hospital admission was unknown.
- The analysis of cumulative use included only the drug that had been used for the longest time at the time of death.

## 1 INTRODUCTION

Clozapine is an effective drug for treatment‐resistant schizophrenia, but has some serious side effects such as agranulocytosis, myocarditis, and ileus.^1^ Since it is also associated with more metabolic side effects than most other antipsychotics,^2^ long‐term use could have a negative impact on life expectancy. Nevertheless, some studies have reported a significant reduction in all‐cause mortality and mortality due to suicide.^3^, ^4^, ^5^, ^6^ The methodology of these studies has been commented.^7^, ^8^ An example is survivor bias, an important source of confounding when studying clozapine and suicide. The risk of suicide is highest in the first years after illness onset,^9^ whereas clozapine is often first prescribed later.^10^ It is difficult to address this confounding effect of time since diagnosis in a prevalence cohort, because a number of patients will have been diagnosed at an unknown point in time before the start of the follow‐up. A single study used an incidence cohort,^11^ but it did not address survivor bias, because the results were not adjusted for duration since onset of illness.

A long observation period is required to study the association between clozapine and cardiovascular mortality, and it is essential to distinguish between the drug used at the time of death (“current use”) and the long‐term use of a drug (often designated “cumulative use”), since patients on clozapine may be switched to another antipsychotic shortly before death. It is also important to adjust the results for somatic comorbidity or treatment, because clinicians may prescribe clozapine more often to patients in better cardiovascular health and because mandatory monitoring may lead to more adequate treatment of somatic disorder. To our knowledge, only two studies on cardiovascular mortality used a measure of cumulative use.^4^, ^12^ The results of these studies showed no differences between cumulative use of clozapine and cumulative use of other antipsychotics, but the results were not adjusted for somatic comorbidity and the treatment thereof.

### 1.1 Aims of the study

In view of the limitations of the above mentioned studies, the aim of the present study was to critically re‐assess the association between clozapine and mortality. We investigated, in a nationwide Danish cohort, whether mortality (all‐cause, due to suicide, or due to cardiovascular disease) associated with *current* use or *cumulative* use of clozapine is lower than that associated with *current* use or *cumulative* use of other categories of antipsychotics, adjusting for somatic comorbidity and the treatment thereof.

## 2 MATERIALS AND METHODS

More details on material and methods are given in Appendix S1.

### 2.1 Data sources

In this multi‐register Danish cohort study, the following databases were linked: (i) The Danish Psychiatric Central Research Register. The computerized registration started on 1 April 1969, and all outpatient contacts were registered after 1994; (ii) The Danish National Prescription Registry for information on dispensed drugs for all Danish inhabitants, from 1995 onwards; (iii) The Causes of Death Register from 1970 to 2015; (iv) The Danish National Patient Registry for somatic health care records from 1977 to 2015; (v) The Danish Civil Registration System from 1973 to 2015. The data that support the findings of this study are available on request from the corresponding author.

### 2.2 Subjects

We defined an incidence cohort, the members of which were all Danish inhabitants aged 15–100 years who were diagnosed with a first non‐affective psychotic disorder (NAPD: ICD‐8 295 and 299; ICD‐10 F20, F25, F28, and F29) between 1 January 1995 and 30 June 2013. Migrants to Denmark were excluded, because information on diagnosis and drug use in the period before immigration was not available. We also defined a prevalence cohort with all individuals ever diagnosed with an NAPD up to 30 June 2013. This cohort included all members of the incidence cohort, plus patients with a first‐registered diagnosis before 1 January 1995 and migrants.

### 2.3 Outcomes

The primary outcome was all‐cause mortality. Secondary outcomes were suicide and cardiovascular mortality.

### 2.4 Exposure

Treatment was categorized as follows: (i) clozapine (reference); (ii) olanzapine; (iii) risperidone; (iv) other Second‐Generation Antipsychotics (SGAs); (v) First Generation Antipsychotics (FGAs); (vi) polypharmacy including clozapine; (vii) polypharmacy not including clozapine; (viii) no antipsychotic medication; (ix) hospital‐delivered antipsychotic, type unknown: antipsychotics are distributed free of charge to patients sentenced to treatment and, since 2008, during the first 2 years subsequent to a diagnosis of schizophrenia. The type of antipsychotic is not known because the drug is not registered in the prescription registry. A small proportion of these patients will not be using any antipsychotic; (x) Drug Unknown: no data available because of hospitalization, inpatient drug use is not registered in the prescription registry. Episodes of drug use were censored on day 15 of hospitalization. We chose this time period because antipsychotic drugs are often continued at the start of a hospitalization and because their effects are likely to last during this period; (xi) no use of antipsychotics.

We conducted separate analyses for current and cumulative use of antipsychotics. Both current and cumulative use are time‐dependent variables and were recalculated at the time of each death event in the cohort, both for the patient who died and for those who were still alive at that time. The currently used antipsychotic was defined as the last drug that was prescribed before a death in the cohort, provided that death occurred after no more than 2 weeks of no use or no more than 2 weeks after hospital admission. Cumulative use was defined as a time‐dependent variable as well and was recalculated at the time of each death in the cohort. For this measure, all episodes of use of a certain antipsychotic were aggregated and the total duration of these episodes was categorized as follows: 0–1, 1–3, 3–6, 6–10 years, and more than 10 years. Thus, one individual could contribute to several monotherapy or polypharmacy categories at different points in time during follow‐up. However, when a death occurred, a subject was placed in only one category of cumulative antipsychotic use, namely, in the category of the drug that had been used the longest at that time. This implies that shorter periods of use of other antipsychotics at this point in time were disregarded. To illustrate this, after consecutively 2 years of olanzapine, 4 years of clozapine, and 3 months of risperidone use, the patient is in the category “risperidone” for the analysis of current use and in the category “clozapine (3–6 years)” for the cumulative use analysis. After 3 months of risperidone, 9 months of olanzapine, and 6 months of clozapine use, a patient is in the “clozapine” category for the analysis of current use and in the category “olanzapine (0–1 year)” for the analysis of cumulative use. Hazard ratios were calculated with the category clozapine use as reference.

### 2.5 Covariates

Baseline variables were age at start of follow‐up, sex, primary psychiatric diagnosis, and psychiatric hospitalization before follow‐up (yes/no). We included the latter variable as a measure of the severity of illness. Duration of illness, that is, duration since first‐registered diagnosis of NAPD at the time of cohort entry, was another baseline variable for members of the prevalence cohort. Time‐dependent variables were substance use disorder, drugs for substance use disorder, mood disorder, use of antidepressants, cardiovascular disorder, drugs for cardiovascular disorders, diabetes, drugs for diabetes, and cancer. The time‐dependent variables changed at the time of their first occurrence and were time‐lasting (permanent). To illustrate this point, after a diagnosis of a mood disorder or the dispension of a drug for cardiovascular problems, this variable remained “yes” for the rest of the follow‐up period.

### 2.6 Statistical analysis

For the main analyses, we used an incidence cohort, because follow‐up can be started at the moment of the first registration of a diagnosis of NAPD. The analyses for cumulative use were conducted in the incidence cohort only, because we did not have information on the use of antipsychotics before the start of follow‐up in the prevalence cohort. Cox proportional hazards regression with time‐dependent variables was used to estimate hazard ratios and 95% confidence intervals (CIs) for the association between exposure to antipsychotics and mortality (all‐cause and cause‐specific). All subjects were followed up from their first diagnosis, their 15th birthday, or from 1 January 1995, whichever occurred last, until death, or 1 July 2014. To allow for the possibility of at least 1 year of follow‐up, the latest entry date was 30 June 2013. Due to violation of the proportional hazards assumption, the Cox analyses were stratified by age at start of follow‐up, sex, Drug Unknown, and in the prevalence cohort also by (registered) duration of illness before the start of the follow‐up. The proportional hazards assumption for the Cox regression models was tested and evaluated by graphical assessment of smoothed hazard estimates plots. Clozapine monotherapy was used as reference. The analyses were performed with Stata. A two‐tailed *p*‐value of <0.05 was considered statistically significant for all tests. Both the analyses of current use and the analyses of cumulative use were conducted for three types of mortality: (i) all‐cause mortality; (ii) mortality due to suicide; and (iii) cardiovascular mortality.

We used two different types of adjustment in order to test the hypothesis that somatic comorbidity and the treatment thereof may influence the association between clozapine and mortality. In the first model, the results were adjusted for the time‐fixed variables age at entry, sex, type of NAPD, and psychiatric hospitalization before start of follow‐up, and for the time‐dependent variables mood disorder, substance use disorder, malignant neoplasms, drugs for mood disorder, and drugs for substance use disorder. We adjusted for type of NAPD and psychiatric hospitalization, because they are proxies for illness severity. We adjusted for malignant neoplasms, in order to make sure that any difference between antipsychotics was not due to the occurrence of neoplasms. In the prevalence cohort, we also adjusted for time since first (registered) NAPD diagnosis. In the second model, the results were also adjusted for cardiovascular and diabetic comorbidity (diagnosis and dispension of drugs) as time‐dependent variables.

### 2.7 Compliance with ethical standards

Statistics Denmark and the Danish Health Data Authority approved use of the data for the study. Ethics approval is not required for retrospective register‐based studies in Denmark.

## 3 RESULTS

### 3.1 Description of cohorts

The incidence and prevalence cohorts consisted of 22,110 patients and 50,881 patients, respectively. Table 1 shows the baseline characteristics. In the 19.5‐year observation period, the mean duration of follow‐up in the incidence cohort was 8.8 years (range 0–19.5; 195 461 person‐years) and in the prevalence cohort 11.3 years (range 0–19.5; 572,617 person‐years). During the follow‐up period, 3612 individuals in the incidence cohort died, 479 due to suicide and 917 from a cardiovascular cause. For the analysis of current use, 375 deaths that occurred after 2 weeks of hospitalization were excluded, resulting in 3237 deaths (407 due to suicide and 851 from a cardiovascular cause). After the exclusion of 1439 individuals who died after 2 weeks of hospitalization, in total 11,948 patients died (1050 deaths due to suicide and 3601 from a cardiovascular cause) in the prevalence cohort. Table S1 shows the number of person‐years and all‐cause and cause‐specific deaths, per category of antipsychotic use, for current use in the incidence cohort. Table S2 shows the numbers of cause‐specific deaths for the categories of cumulative use in the incidence cohort. There was a large difference in the number of suicides associated with current use of clozapine (Table S1, *N* = 5) and cumulative use of clozapine up to 1 year (Table S2, *N* = 113).

**TABLE 1:** Baseline characteristics of all people in Denmark first diagnosed with a non‐affective psychotic disorder between 1 January 1995 and 1 July 2013 (incidence cohort) and of all people ever diagnosed with a non‐affective psychotic disorder (prevalence cohort).

	Incidence cohort *N* = 22,110	Prevalence cohort *N* = 50,881
Male	53.4%	55.6%
Age at inclusion in cohort (SD)	35.7 (16.4)	39.7 (16.5)
Age of first diagnosis of psychosis (SD)	35.7 (16.4)	34.1 (14.7)
Diagnosis at start follow‐up		
Schizophrenia (ICD−8 295)	N.A.	15,310 (30.1%)
Schizophrenia (ICD−10 F20)	16,611 (75.1%)	23,627 (46.4%)
Schizoaffective disorders (ICD−10 F25)	2076 (9.4%)	2926 (5.8%)
Unspecified psychosis (ICD−8 299)	N.A.	3946 (7.8%)
Other psychotic disorder not due to a substance or known physiological condition (ICD−10 F28)	926 (4.2%)	1393 (2.7%)
Unspecified psychosis not due to a substance or known physiological condition (ICD−10 F29)	2497 (11.3%)	3679 (7.2%)
Diagnosis at end of follow‐up		
Schizophrenia (ICD−8 295)	N.A	16,384 (32.2%)
Schizophrenia (ICD−10 F20)	17,994 (81.4%)	26,163 (51.4%)
Schizoaffective disorders (ICD−10 F25)	1660 (7.5%)	2395 (4.7%)
Unspecified psychosis (ICD−8 299)	N.A.	2360 (4.6%)
Other psychotic disorder not due to a substance or known physiological condition (ICD−10 F28)	702 (3.2%)	1067 (2.1%)
Unspecified psychosis not due to a substance or known physiological condition (ICD−10 F29)	1754 (7.9%)	2513 (4.9%)
Mood disorder	5360 (24.2%)	10,855 (21.3%)
Psychiatric Hospitalization	16,365 (74.0%)	42,357 (83.3%)
Substance use disorder	6014 (27.2%)	12,352 (24.3%)
Drugs for substance use disorder	554 (2.5%)	668 (1.3%)
Antidepressant drug	6102 (27.6%)	7702 (15.1%)
Diabetes mellitus	549 (2.5%)	1185 (2.3%)
Circulatory system diseases	2062 (9.3%)	3992 (7.9%)
Malignant Neoplasms	438 (2.0%)	1055 (2.1%)

### 3.2 Current use

#### 3.2.1 All‐cause mortality

**FIGURE 1:**
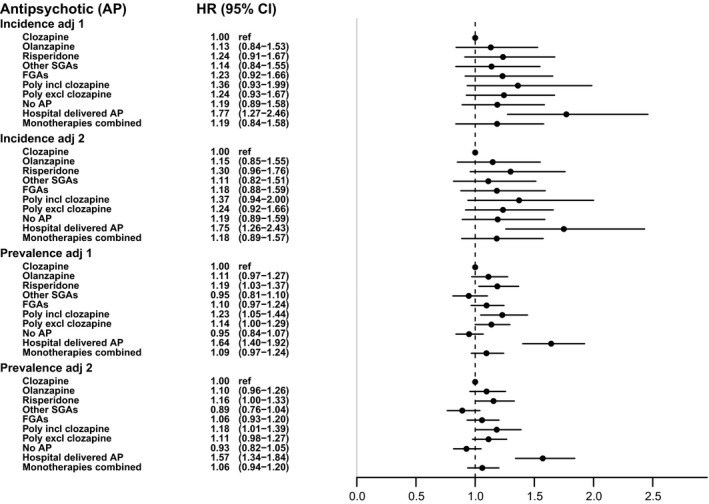
Adjusted hazard ratios for all‐cause mortality during current use of antipsychotics compared to current use of clozapine (reference), between January 1995 and July 2014, in Danish incidence and prevalence cohorts of patients with a non‐affective psychotic disorder. Adj 1: Adjusted for age, sex, type of non‐affective psychotic disorder, mood disorder, substance use disorder, psychiatric hospitalization, and in the prevalence cohort for duration of illness. Adj 2: Also adjusted for somatic comorbidity and the treatment of somatic disorders.

Figure 1 shows the results for the incidence and the prevalence cohorts, with two types of adjustment. In the incidence cohort, clozapine was not associated with a lower mortality, except when compared to hospital‐delivered antipsychotics. In the prevalence cohort, although the HRs were closer to 1 than in the incidence cohort, the confidence intervals were smaller and the HRs for risperidone, polypharmacy including clozapine, and hospital‐delivered antipsychotics were significantly higher than that for clozapine (monotherapy). The figure shows that the differences in results between the two types of adjustment were very small. The additional analysis with all monotherapies combined (the last bar in the figure) showed no significant effect of clozapine in either cohort.

#### 3.2.2 Suicide

**FIGURE 2:**
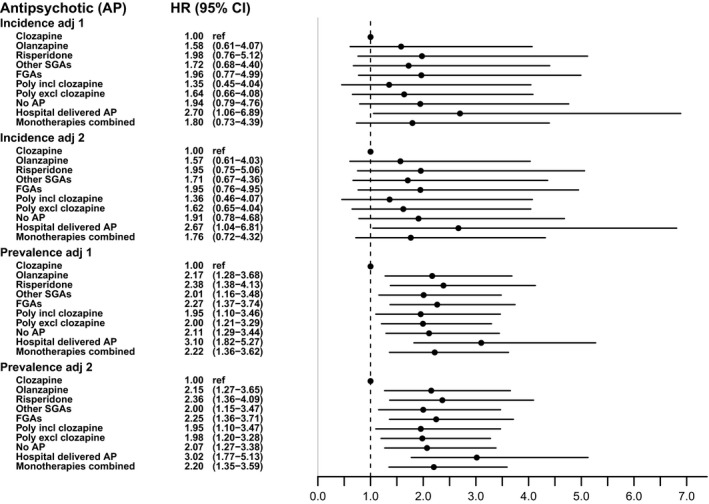
Adjusted hazard ratios for mortality due to suicide during current use of antipsychotics compared to current use of clozapine (reference), between January 1995 and July 2014, in Danish incidence and prevalence cohorts of patients with a non‐affective psychotic disorder. The observation period was from January 1995 to July 2014. Adj 1: Adjusted for age, sex, type of non‐affective psychotic disorder, mood disorder, substance use disorder, psychiatric hospitalization, and in the prevalence cohort for duration of illness. Adj 2: Also adjusted for somatic comorbidity and the treatment of somatic disorders.

Table S1 shows that none of the clozapine users in the incidence cohort died by suicide in 6 years after the first diagnosis (during 706 + 1017 = 1723 person‐years). The results of the analyses for current antipsychotic use are shown in Figure 2. In the incidence cohort, mortality due to suicide during the use of clozapine was lower compared to that during the use of other antipsychotics, but this difference was only significant for the comparison with users of hospital‐delivered antipsychotics. In the prevalence cohort, all categories of current antipsychotic use were associated with a significantly higher mortality compared to clozapine. These differences were larger than in the incidence cohort, and the confidence intervals were smaller. Accordingly, mortality for all monotherapies combined was significantly higher compared to clozapine in the prevalence cohort (HR_adj_ = 2.20; 95% CI 1.35–3.59), but not in the incidence cohort (HR_adj_ = 1.76; 95% CI 0.72–4.32). Adjustment for somatic comorbidity did not affect the results.

#### 3.2.3 Cardiovascular mortality

**FIGURE 3:**
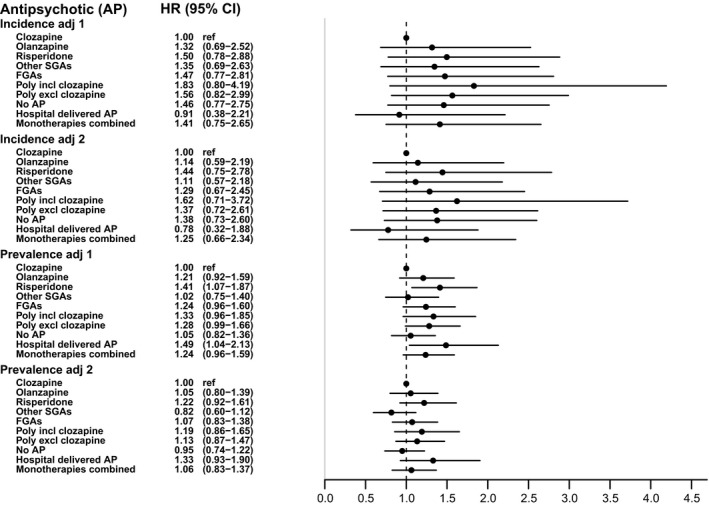
Adjusted hazard ratios for cardiovascular mortality during current use of antipsychotics compared to current use of clozapine (reference), between January 1995 and July 2014, in Danish incidence and prevalence cohorts of patients with a non‐affective psychotic disorder. The observation period was from January 1995 to July 2014. Adj 1: Adjusted for age, sex, type of non‐affective psychotic disorder, mood disorder, substance use disorder, psychiatric hospitalization, and in the prevalence cohort for duration of illness. Adj 2: Also adjusted for somatic comorbidity and the treatment of somatic disorders.

In the incidence cohort, the hazard of cardiovascular mortality was lower with clozapine than with most other antipsychotics, but the confidence intervals were broad and included 1 (Figure 3). As for the prevalence cohort, cardiovascular mortality associated with clozapine was significantly lower in the first adjusted model than that associated with risperidone and hospital‐delivered antipsychotics. However, after adjustment for cardiovascular and diabetic comorbidity, the HRs slightly decreased toward 1 and were no longer statistically significant.

### 3.3 Cumulative use

#### 3.3.1 All‐cause mortality

**FIGURE 4:**
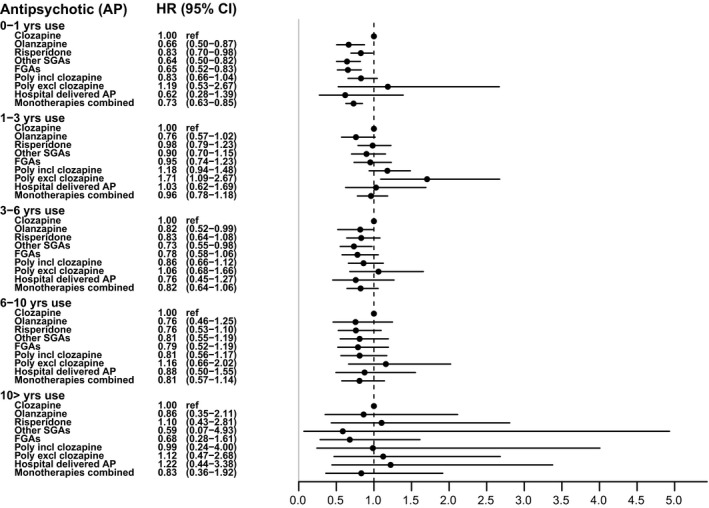
Adjusted hazard ratios for all‐cause mortality after cumulative use of antipsychotics compared to cumulative use of clozapine (reference), between January 1995 and July 2014, in a Danish incidence cohort of patients with a non‐affective psychotic disorder. Comparisons with clozapine were done within each of the groups distinguished by the same length of antipsychotic use: 0–1, 1–3, 3–6, 6–10 years, and more than 10 years. The observation period was from January 1995 to July 2014. Adjusted for age, sex, type of non‐affective psychotic disorder, mood disorder, substance use disorder, psychiatric hospitalization, somatic comorbidity, and the treatment of somatic disorders.

Figure 4 shows that cumulative use of clozapine for up to 1 year was associated with a higher all‐cause mortality than cumulative use of most other antipsychotics. This difference cannot be attributed to myocarditis, because there were no cases during this 1‐year period. This difference in all‐cause mortality was not present after longer use of the drugs. Adjustment for somatic comorbidity had a minimal effect (figure with first type of adjustment not shown).

#### 3.3.2 Suicide

The fully adjusted analysis of cumulative use also shows a significantly higher suicide rate among patients who had used clozapine for up to 1 year than among patients who had used polypharmacy including clozapine, olanzapine, other SGAs, and all monotherapies combined for a similar time (see Figure S1). After cumulative use of more than 1 year, this difference in mortality due to suicide was no longer significant. Of the individuals who committed suicide, 24% had used clozapine monotherapy for longer than any other antipsychotic during a period up to 1 year (113 of 479 suicides) (Table S2).

#### 3.3.3 Cardiovascular deaths

Except for cumulative use of polypharmacy excluding clozapine (1–3 years), there were no significant differences between use of clozapine and use of other antipsychotics in cardiovascular mortality, irrespective of the duration of use or adjustment for somatic comorbidity (see Figure S2).

## 4 DISCUSSION

Our first objective was to investigate whether mortality (all‐cause and cause‐specific) associated with “current” use of clozapine was lower than that associated with “current” use of other antipsychotics. A second objective was to assess whether findings were similar when “cumulative” use was analyzed instead of “current” use. In general, current use of clozapine was not associated with a lower all‐cause or cardiovascular mortality in the incidence or prevalence cohort. In both cohorts, however, the numbers of suicides were lower with current use of clozapine. Remarkably, cumulative use of clozapine for 0–1 year was associated with a higher mortality (all‐cause and suicide) compared to cumulative use of almost all other categories. Cumulative use of clozapine for 6 years or longer was not associated with a higher all‐cause or cardiovascular mortality, despite its metabolic side effects. Adjustment for the presence and treatment of somatic comorbidity hardly affected the results.

### 4.1 Interpretation and comparison with other studies

#### 4.1.1 Current use

Our finding of no significantly lower all‐cause mortality among users of clozapine is consistent with several other large database studies,^12^, ^13^, ^14^, ^15^ but not with the study that most closely resembled our study in design, the FIN11 study.^4^ It is possible that results in different countries vary due to differences in prescription rates (more or less restrictive use) or differences in the National registers that were used. However, the FIN20 study yielded different results than the FIN11 study,^4^ despite similar inclusion criteria and year of start of follow‐up. See Table S3 for differences and similarities between the Finnish studies and the present study. The FIN11 study reported a large reduction of mortality among users of this drug, for all‐cause and cause‐specific mortality. As already pointed out by de Hert et al.,^7^ the authors disregarded two‐thirds of all deaths, by excluding deaths after a hospitalization of more than 2 days. In the FIN20 study,^12^ follow‐up time was censored after 7 days of hospital admission instead of 2 days. The results of the FIN20 study show that clozapine was no longer associated with a significantly lower mortality compared to all other categories of antipsychotics. If these different findings of the Finnish studies are partly caused by the difference in censoring deaths during hospital admission, this may also explain the differences in results between our study and the FIN11 study, given that we excluded deaths after 14 days of hospitalization. In two studies that were restricted to treatment‐resistant patients,^16^, ^17^ a significantly lower mortality associated with clozapine use was found. However, mortality was only significantly lower when comparing the use of clozapine to non‐use of clozapine (including no use of any antipsychotic).

Our finding of a lower suicide rate during the use of clozapine is in line with the results of many previous studies.^3^, ^4^, ^5^, ^6^, ^12^, ^18^ The differences in hazard ratios for current use between the incidence and the prevalence cohorts in our study were slightly smaller than expected, given the possible survivor bias in the prevalence cohort. The HRadj for mortality due to suicide during use of all monotherapies combined dropped from 2.20 (CI 1.35–3.59) in the prevalence cohort to 1.76 (CI 0.72–4.32) in the incidence cohort, but the confidence intervals were broad and overlapping. In a sensitivity analysis of the FIN20 study, excluding the first ten years of follow‐up, clozapine dropped from third to seventh place in the ranking of the most beneficial antipsychotics and all‐cause mortality. Thus, survivorship bias may have influenced the results of the FIN20 study.

#### 4.1.2 Cumulative use

There are two studies to which we can compare our results on cumulative use, despite different designs and statistical analyses. The first is the FIN11 study,^4^ which also compared cumulative use of different categories of antipsychotics. In this study, a prevalence cohort was used, while we used an incidence cohort with complete information on drug use before start follow‐up. In addition, the analysis strategies differed. In the FIN11 study, all episodes of use were included in the analysis and relative risks were calculated. In such an analysis, some people can be in two different categories at a certain time point and may have been compared with themselves. In our Cox regression analyses, patients were put in exclusive categories at each time point and, thus, were never compared to themselves at a single time point. Consequently, they were in the category of the drug they had used for the longest time and the use of other antipsychotics was disregarded. The second study, the FIN20 study,^12^ compared cumulative use of clozapine only to cumulative use of any antipsychotic and calculated odds ratios. Neither the FIN11 nor the FIN20 reported significant differences in cardiovascular mortality between cumulative use of clozapine and cumulative use of other antipsychotics. This is in line with the results on cumulative use of our study.

Remarkably, cumulative use of clozapine for more than 6 years did not seem to increase cardiovascular mortality, despite its metabolic side effects. This is in line with the findings of Kelly et al.,^19^ who did not find significant differences in cardiovascular mortality between those who started clozapine and those who started risperidone after a 6‐ to 10‐year observation period. This may be explained by the effectiveness of clozapine against psychotic symptoms and the subsequent reduction of stress and substance abuse.

#### 4.1.3 Current and cumulative use combined

Our findings of higher overall mortality and higher suicide mortality among patients who had used clozapine for up to 1 year, in combination with the lower mortality during current use of this drug, suggest that the extra deaths occurred after the termination of clozapine use. Concerning suicide, Tables S1 and S2 show that only 5 suicides occurred during clozapine use, while 113 suicides occurred after 0–1 year of cumulative clozapine use. This finding is in line with the previous report from Denmark^16^ of an increased risk of death after termination versus during clozapine use (HR 2.65, 95% CI 1.47–4.78). The authors of this report deemed it likely that the excess mortality rate in the first year or even within 3 months of discontinuation was due to causes other than suicide, because clozapine could have been discontinued due to severe medical conditions related or unrelated to clozapine treatment. However, we do find an increased risk of suicide after clozapine discontinuation, a phenomenon previously described by Patchan et al.^20^ and Walker et al.^3^ Walker et al.^3^ reported a markedly decreased suicide rate during current use compared to past use of clozapine (standardized mortality ratio 0.17; CI 0.10–0.30). Their conclusion that clozapine decreases the suicide rate among users is only half the story, because we found that the suicide rate associated with cumulative clozapine use was higher than that for cumulative use of other antipsychotics. As clozapine is a drug of last resort, stopping it may lead to relapse or give rise to despair and an increased suicidality. Furthermore, since clozapine is indicated for suicidal patients, such patients are more likely to start using clozapine (ie, confounding by indication). This could contribute to the high risk of suicide after termination of this drug. There is no good evidence that the increased risk of suicide after termination is due to a rebound effect or to acute clozapine withdrawal.

### 4.2 Strengths and limitations

To our knowledge, this is the first large study to compare mortality associated with cumulative use of different antipsychotics using a representative incidence cohort with complete information on medication use from the start of treatment. In addition, the incidence cohort of 22 110 patients is larger than in any other study on clozapine and mortality. With a maximum of 19.5 years, the follow‐up time of the cohort was very long. We included 90% of all deaths in the analyses of current use, and this is the first study to adjust the results for somatic comorbidity and somatic treatment. To ensure that differences in findings between our study and other studies are not due to the use of an incidence cohort, we repeated our analysis in a prevalence cohort.

Several limitations need to be mentioned. First, the databases we used did not include direct information on disease severity or lifestyle factors. Second, there was no information on medication use during hospitalization, as a result of which we had to exclude deaths during longer hospitalizations. Third, the prescription database consists of dispensed drugs, and it is unknown whether or not the patients have actually used the drugs. Fourth, psychiatric registrations before 1969 were incomplete, leading to missing information about age at disease onset for many patients in the prevalence cohort and thus to residual confounding. Fifth, despite the long observation period (19.5 years), the power of the analyses of cause‐specific mortality in the incidence cohort was not optimal. Sixth, the category “non‐users of antipsychotics” is likely to consist of patients with mild symptoms not needing antipsychotics and patients with more severe symptoms who refuse treatment. We were not able to distinguish between these groups. Seventh, we did not correct for multiple testing. Since almost none of our results were significant, a correction for multiple testing would not have changed this result. Eighth, in the incidence cohort analyses, we excluded migrants because information on diagnoses and antipsychotic use before migrating was unknown. Ninth, because the follow‐up started at the moment of first diagnosis of NAPD, previous psychotropic and antipsychotic drug use was disregarded. Tenth, our definition of cumulative use does not take into account whether this drug has been discontinued and the length of time since discontinuation. Finally, there may have been residual confounding. Since clozapine is more commonly prescribed to severely ill patients, we adjusted for type of diagnosis and history of hospitalization. We do not know whether this adjustment was adequate, because it hardly changed the results. More adequate adjustments for severity of illness could therefore have led to more favorable outcomes for clozapine.

To conclude, we found no major differences in mortality between users of clozapine and users of other antipsychotics. This should encourage clinicians to prescribe clozapine to the patients who need it. Moreover, our findings add to an increasing body of evidence that clozapine use protects against suicide. This protective effect is lost when clozapine is discontinued. Clinicians should carefully monitor patients from whom clozapine has been withdrawn or consider a clozapine re‐challenge.

## DECLARATION OF INTERESTS

None.

### PEER REVIEW

The peer review history for this article is available at https://publons.com/publon/10.1111/acps.13267.

## Supporting information

Figure S1

Figure S2

Table S1

Table S2

Table S3

Appendix S1

## Data Availability

The data are not publicly available due to privacy or ethical restrictions.
