# Supplementary material for: Clozapine and mortality: A comparison with other antipsychotics in a nationwide Danish cohort study
Source: Acta Psychiatr Scand. 2020 Dec 25;143(3):216–26. doi: 10.1111/acps.13267 (PMC7986383; doi:10.1111/acps.13267)

| Antipsychotic (AP) | HR (95% CI) | |
|---|---|---|
| **0−1 yrs use** | | |
| Clozapine | 1.00 | ref |
| Olanzapine | 0.53 | (0.29−0.96) |
| Risperidone | 0.65 | (0.42−1.01) |
| Other SGAs | 0.38 | (0.19−0.75) |
| FGAs | 0.74 | (0.42−1.29) |
| Poly incl clozapine | 0.54 | (0.32−0.90) |
| Poly excl clozapine | 0.52 | (0.07−3.75) |
| Hospital delivered AP | NA | ( NA− NA) |
| Monotherapies combined | 0.65 | (0.46−0.91) |
| **1−3 yrs use** | | |
| Clozapine | 1.00 | ref |
| Olanzapine | 0.52 | (0.25−1.11) |
| Risperidone | 0.96 | (0.55−1.68) |
| Other SGAs | 0.97 | (0.54−1.74) |
| FGAs | 1.35 | (0.76−2.41) |
| Poly incl clozapine | 0.64 | (0.36−1.14) |
| Poly excl clozapine | 0.28 | (0.04−2.07) |
| Hospital delivered AP | NA | ( NA− NA) |
| Monotherapies combined | 0.95 | (0.60−1.48) |
| **3−6 yrs use** | | |
| Clozapine | 1.00 | ref |
| Olanzapine | 0.50 | (0.21−1.18) |
| Risperidone | 0.84 | (0.44−1.60) |
| Other SGAs | 0.58 | (0.28−1.21) |
| FGAs | 0.43 | (0.17−1.06) |
| Poly incl clozapine | 0.81 | (0.46−1.43) |
| Poly excl clozapine | 0.58 | (0.20−1.69) |
| Hospital delivered AP | 0.86 | (0.30−2.52) |
| Monotherapies combined | 0.68 | (0.40−1.15) |
| **6−10 yrs use** | | |
| Clozapine | 1.00 | ref |
| Olanzapine | 0.36 | (0.04−2.98) |
| Risperidone | 0.57 | (0.16−2.03) |
| Other SGAs | 0.73 | (0.22−2.34) |
| FGAs | 0.48 | (0.10−2.38) |
| Poly incl clozapine | 0.97 | (0.37−2.55) |
| Poly excl clozapine | 1.75 | (0.50−6.17) |
| Hospital delivered AP | 0.57 | (0.07−4.70) |
| Monotherapies combined | 0.62 | (0.25−1.56) |

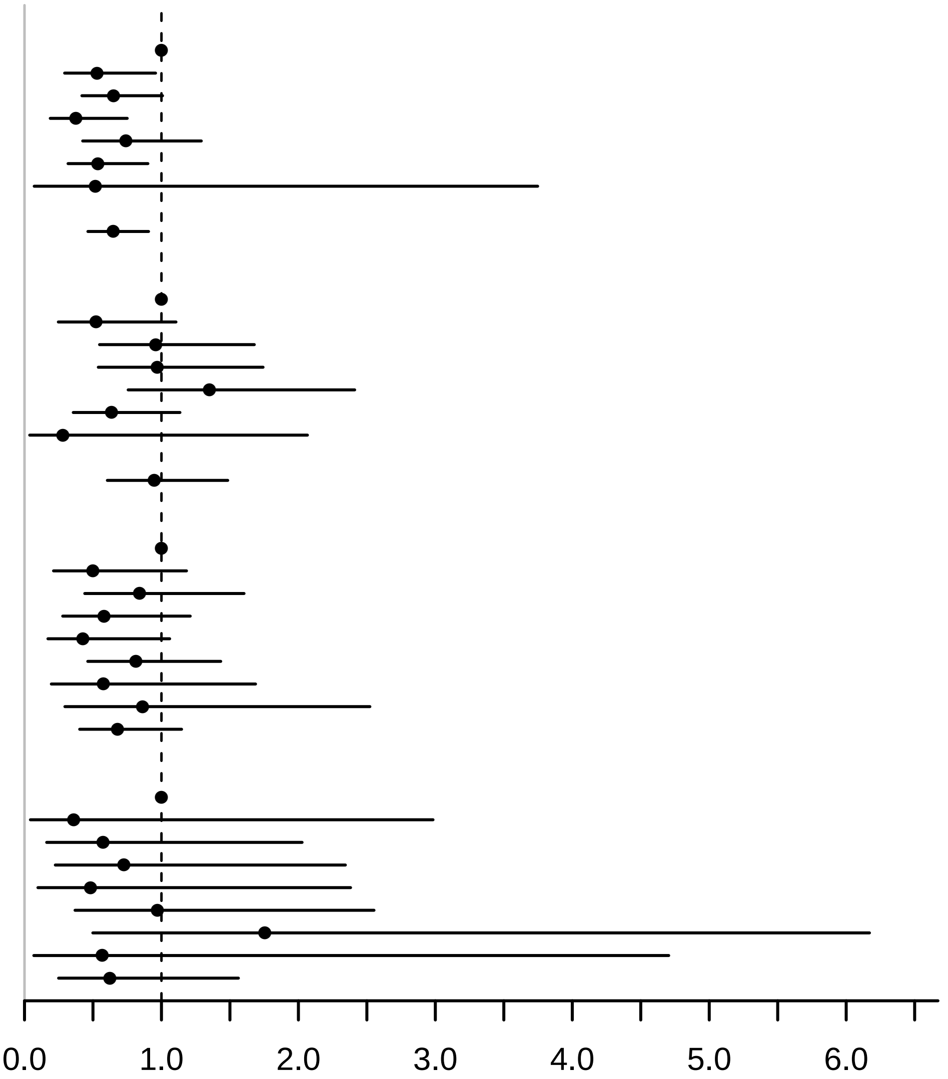

Supplement: Supplementary file 1 — Figure S1 [file ACPS-143-216-s005.pdf]
