# Supplementary material for: Clozapine and mortality: A comparison with other antipsychotics in a nationwide Danish cohort study
Source: Acta Psychiatr Scand. 2020 Dec 25;143(3):216–26. doi: 10.1111/acps.13267 (PMC7986383; doi:10.1111/acps.13267)

Antipsychotic (AP)	HR (95% CI)	
**0−1 yrs use**		
Clozapine	1.00	ref
Olanzapine	0.90	(0.54−1.53)
Risperidone	1.00	(0.70−1.42)
Other SGAs	0.68	(0.42−1.11)
FGAs	0.72	(0.45−1.17)
Poly incl clozapine	0.84	(0.52−1.39)
Poly excl clozapine	1.00	(0.14−7.28)
Hospital delivered AP	2.09	(0.83−5.24)
Monotherapies combined	0.83	(0.60−1.14)
**1−3 yrs use**		
Clozapine	1.00	ref
Olanzapine	0.74	(0.36−1.49)
Risperidone	1.32	(0.77−2.25)
Other SGAs	1.13	(0.63−2.02)
FGAs	1.35	(0.75−2.42)
Poly incl clozapine	1.53	(0.87−2.69)
Poly excl clozapine	3.64	(1.50−8.83)
Hospital delivered AP	1.23	(0.41−3.66)
Monotherapies combined	1.19	(0.70−2.00)
**3−6 yrs use**		
Clozapine	1.00	ref
Olanzapine	0.82	(0.39−1.74)
Risperidone	0.97	(0.52−1.82)
Other SGAs	1.13	(0.59−2.17)
FGAs	1.18	(0.60−2.30)
Poly incl clozapine	0.74	(0.38−1.45)
Poly excl clozapine	1.64	(0.61−4.40)
Hospital delivered AP	0.24	(0.03−1.85)
Monotherapies combined	1.14	(0.60−2.18)
**6−10 yrs use**		
Clozapine	1.00	ref
Olanzapine	0.78	(0.28−2.15)
Risperidone	1.11	(0.50−2.43)
Other SGAs	0.57	(0.24−1.36)
FGAs	0.63	(0.25−1.57)
Poly incl clozapine	0.81	(0.36−1.83)
Poly excl clozapine	0.57	(0.12−2.75)
Hospital delivered AP	0.23	(0.03−1.84)
Monotherapies combined	1.00	(0.45−2.23)

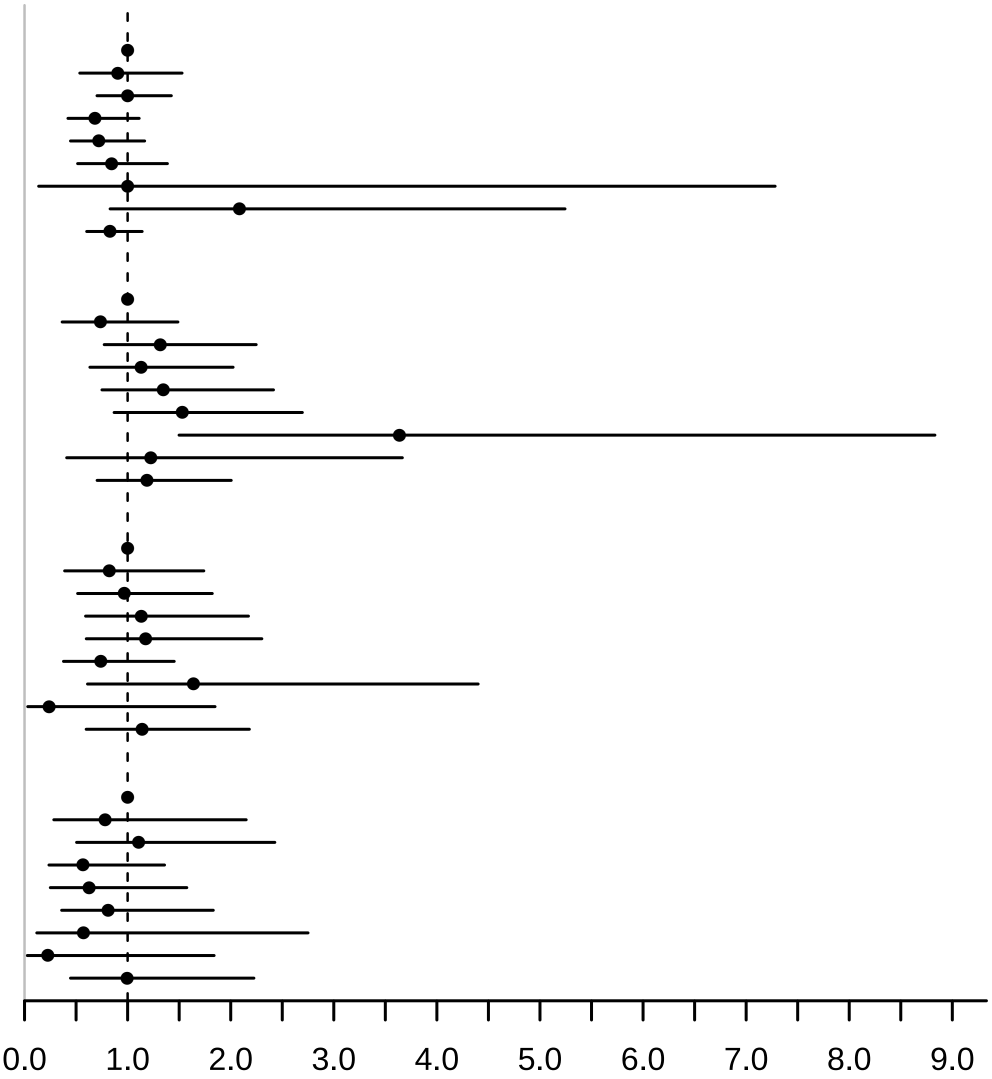

Supplement: Supplementary file 2 — Figure S2 [file ACPS-143-216-s004.pdf]
