# Supplementary material for: Clozapine and mortality: A comparison with other antipsychotics in a nationwide Danish cohort study
Source: Acta Psychiatr Scand. 2020 Dec 25;143(3):216–26. doi: 10.1111/acps.13267 (PMC7986383; doi:10.1111/acps.13267)
Supplement: Supplementary file 3 — Table S1 [file ACPS-143-216-s003.docx]

Table S1 Patient-years, all-cause and cause-specific deaths per category of antipsychotic used at the time of death, by length of follow-up: 0-3, 3-6 and more than 6 years. Data derived from an incidence cohort consisting of all people in Denmark first diagnosed with a non-affective psychotic disorder between 1 January 1995 and 1 July 2013).

|  | Follow-up time (years), years after first diagnosis | N* | Patient-years | Allcause events | Allcause events  /1000patient-years | Suicide events | Suicide events /1000patient-years | Cardiovascular events | Cardiovascular events /1000patient-years |
| --- | --- | --- | --- | --- | --- | --- | --- | --- | --- |
|  |  | 22,110 | 195,461 | 3,612 | 18.5 | 407 | 2.1 | 851 | 4.4 |
|  |  |  |  |  |  |  |  |  |  |
| Clozapine | total | 1,677 | 4,112 | 49 | 11.9 | 5 | 1.2 | 10 | 2.4 |
|  | 0_3 | 878 | 706 | 10 | 14.2 | 0 | 0.0 | 4 | 5.7 |
|  | 3_6 | 921 | 1,017 | 13 | 12.8 | 0 | 0.0 | 3 | 2.9 |
|  | >6 | 957 | 2,388 | 26 | 10.9 | 5 | 2.1 | 3 | 1.3 |
|  |  |  |  |  |  |  |  |  |  |
| No Antipsychotics | total | 21,340 | 71,586 | 1,065 | 14.9 | 152 | 2.1 | 282 | 3.9 |
|  |  |  |  |  |  |  |  |  |  |
|  | 0_3 | 20,745 | 23,265 | 343 | 14.7 | 79 | 3.4 | 95 | 4.1 |
|  | 3_6 | 15,464 | 17,513 | 229 | 13.1 | 26 | 1.5 | 65 | 3.7 |
|  | >6 | 12,141 | 30,807 | 493 | 16.0 | 47 | 1.5 | 122 | 4.0 |
|  |  |  |  |  |  |  |  |  |  |
| Unknown AP (hospital delivered) | total | 5,422 | 7,876 | 141 | 17.9 | 39 | 5.0 | 10 | 1.3 |
|  | 0_3 | 4,546 | 2,767 | 49 | 17.7 | 12 | 4.3 | 3 | 1.1 |
|  | 3_6 | 1,276 | 1,921 | 29 | 15.1 | 11 | 5.7 | 2 | 1.0 |
|  | >6 | 1,179 | 3,187 | 63 | 19.8 | 16 | 5.0 | 5 | 1.6 |
|  |  |  |  |  |  |  |  |  |  |
| Polypharmacy excluding clozapine | total | 11,032 | 28,976 | 494 | 17.0 | 63 | 2.2 | 125 | 4.3 |
|  | 0_3 | 8,149 | 7,872 | 144 | 18.3 | 22 | 2.8 | 39 | 5.0 |
|  | 3_6 | 5,908 | 7,203 | 90 | 12.5 | 15 | 2.1 | 23 | 3.2 |
|  | >6 | 5,289 | 13,900 | 260 | 18.7 | 26 | 1.9 | 63 | 4.5 |
|  |  |  |  |  |  |  |  |  |  |
| Polypharmacy including clozapine | total | 1,723 | 5,267 | 61 | 11.6 | 9 | 1.7 | 13 | 2.5 |
|  | 0_3 | 895 | 755 | 7 | 9.3 | 0 | 0.0 | 3 | 4.0 |
|  | 3_6 | 963 | 1,257 | 16 | 12.7 | 2 | 1.6 | 5 | 4.0 |
|  | >6 | 1,043 | 3,254 | 38 | 11.7 | 7 | 2.2 | 5 | 1.5 |
|  |  |  |  |  |  |  |  |  |  |
| Olanzapine | total | 6,951 | 16,947 | 369 | 21.8 | 32 | 1.9 | 103 | 6.1 |
|  | 0_3 | 5,022 | 4,633 | 98 | 21.2 | 14 | 3.0 | 30 | 6.5 |
|  | 3_6 | 3,181 | 4,186 | 74 | 17.7 | 5 | 1.2 | 19 | 4.5 |
|  | >6 | 2,878 | 8,128 | 197 | 24.2 | 13 | 1.6 | 54 | 6.6 |
|  |  |  |  |  |  |  |  |  |  |
| Risperidone | total | 6,553 | 13,454 | 314 | 23.3 | 29 | 2.2 | 95 | 7.1 |
|  | 0_3 | 4,731 | 4,086 | 89 | 21.8 | 12 | 2.9 | 24 | 5.9 |
|  | 3_6 | 2,736 | 3,389 | 84 | 24.8 | 7 | 2.1 | 35 | 10.3 |
|  | >6 | 2,470 | 5,978 | 141 | 23.6 | 10 | 1.7 | 36 | 6.0 |
|  |  |  |  |  |  |  |  |  |  |
| First generation antipsychotics | total | 9,813 | 18,820 | 492 | 26.1 | 42 | 2.2 | 150 | 8.0 |
|  | 0_3 | 8,119 | 6,492 | 178 | 27.4 | 17 | 2.6 | 66 | 10.2 |
|  | 3_6 | 4,506 | 4,636 | 99 | 21.4 | 9 | 1.9 | 22 | 4.7 |
|  | >6 | 3,825 | 7,691 | 215 | 28.0 | 16 | 2.1 | 62 | 8.1 |
|  |  |  |  |  |  |  |  |  |  |
| Other second generation antipsychotics | total | 10,417 | 19,164 | 252 | 13.1 | 36 | 1.9 | 63 | 3.3 |
|  | 0_3 | 6,612 | 5,489 | 62 | 11.3 | 14 | 2.6 | 17 | 3.1 |
|  | 3_6 | 4,724 | 4,783 | 53 | 11.1 | 11 | 2.3 | 13 | 2.7 |
|  | >6 | 4,826 | 8,891 | 137 | 15.4 | 11 | 1.2 | 33 | 3.7 |

* Every person can contribute to multiple categories of antipsychotic use.
