# Supplementary material for: Clozapine and mortality: A comparison with other antipsychotics in a nationwide Danish cohort study
Source: Acta Psychiatr Scand. 2020 Dec 25;143(3):216–26. doi: 10.1111/acps.13267 (PMC7986383; doi:10.1111/acps.13267)
Supplement: Supplementary file 4 — Table S2 [file ACPS-143-216-s002.docx]

Table S2. Suicides and cardiovascular deaths in each category of cumulative use of antipsychotics in incidence cohort (all people in Denmark first diagnosed with a non-affective psychotic disorder between 1 January 1995 and 1 July 2013).

| Use during | Type of antipsychotic therapy | Suicides | Cardiovascular deaths |
| --- | --- | --- | --- |
|  |  |  |  |
|  | never used an antipsychotic drug | 14 | 38 |
| 0-1 year | clozapine monotherapy | 113 | 74 |
| 0-1 year | clozapine polytherapy | 18 | 23 |
| 0-1 year | olanzapine monotherapy | 13 | 19 |
| 0-1 year | risperidon monotherapy | 28 | 69 |
| 0-1 year | First generation antipsychotics monotherapy | 15 | 24 |
| 0-1 year | other second generation antipsychotics monotherapy | 9 | 23 |
| 0-1 year | polytherapy excluding clozapine | 1 | 1 |
| 0-1 year | unknown antipsychotic | 0 | 5 |
|  |  |  |  |
| 1-3 years | clozapine monotherapy | 34 | 19 |
| 1-3 years | clozapine polytherapy | 19 | 44 |
| 1-3 years | olanzapine monotherapy | 9 | 16 |
| 1-3 years | risperidon monotherapy | 22 | 80 |
| 1-3 years | First generation antipsychotics monotherapy | 19 | 38 |
| 1-3 years | other second generation antipsychotics monotherapy | 18 | 38 |
| 1-3 years | polytherapy excluding clozapine | 1 | 7 |
| 1-3 years | unknown antipsychotic | 0 | 4 |
|  |  |  |  |
| 3-6 years | clozapine monotherapy | 23 | 13 |
| 3-6 years | clozapine polytherapy | 29 | 32 |
| 3-6 years | olanzapine monotherapy | 7 | 17 |
| 3-6 years | risperidon monotherapy | 18 | 64 |
| 3-6 years | First generation antipsychotics monotherapy | 6 | 33 |
| 3-6 years | other second generation antipsychotics monotherapy | 11 | 44 |
| 3-6 years | polytherapy excluding clozapine | 4 | 6 |
| 3-6 years | unknown antipsychotic | 4 | 1 |
|  |  |  |  |
| 6-10 years | clozapine monotherapy | 7 | 8 |
| 6-10 years | clozapine polytherapy | 12 | 31 |
| 6-10 years | olanzapine monotherapy | 1 | 8 |
| 6-10 years | risperidon monotherapy | 4 | 58 |
| 6-10 years | First generation antipsychotics monotherapy | 2 | 14 |
| 6-10 years | other second generation antipsychotics monotherapy | 5 | 20 |
| 6-10 years | polytherapy excluding clozapine | 4 | 2 |
| 6-10 years | unknown antipsychotic | 1 | 1 |
|  |  |  |  |
| >10 years | clozapine monotherapy | 0 | 0 |
| >10 years | clozapine polytherapy | 0 | 2 |
| >10 years | olanzapine monotherapy | 2 | 6 |
| >10 years | risperidon monotherapy | 0 | 6 |
| >10 years | First generation antipsychotics monotherapy | 0 | 15 |
| >10 years | other second generation antipsychotics monotherapy | 0 | 0 |
| >10 years | polytherapy excluding clozapine | 1 | 14 |
| >10 years | unknown antipsychotic | 5 | 0 |
|  |  |  |  |
| Total |  | 479 | 917 |
