# Supplementary material for: Clozapine and mortality: A comparison with other antipsychotics in a nationwide Danish cohort study
Source: Acta Psychiatr Scand. 2020 Dec 25;143(3):216–26. doi: 10.1111/acps.13267 (PMC7986383; doi:10.1111/acps.13267)
Supplement: Supplementary file 5 — Table S3 [file ACPS-143-216-s006.docx]

Table S3 Comparison between the FIN11 study (Tiihonen et al, 2009), the FIN20 study (Taipale et al, 2020) and the present Danish study (Zalm et al.).

|  | FIN11 study | FIN20 study | | Present study | |
| --- | --- | --- | --- | --- | --- |
| Authors | Tiihonen et al. | Taipale et al. | | Zalm et al. | |
| Year of publication | 2009 | 2020 | |  | |
| Country | Finland | Finland | | Denmark | |
| Type of cohort | prevalence | prevalence | incidence | prevalence | incidence |
| Included ICD diagnoses | ICD-8: 295, except 295.7  ICD-9: 2951, 2952, 2953, 2954A, 2956, 2957A, 2959;  ICD-10: F20, F21, F25 | ICD-8 and ICD-9: 295  ICD-10: F20, F25 | ICD-10: F20, F25 | ICD 8: 295, 299  ICD 10: F20, F25, F28, F29 | ICD 10: F20, F25, F28, F29 |
| Diagnoses included from | 1973 to 2004 | 1972 to2014 | January 1^st^ 1996 to 2014 | 1969 to July 1st 2013 | January 1st 1995 to July 1st 2013 |
| Start follow up | January 1^st^ 1996 or at discharge after first stay in hospital for schizophrenia. | January 1^st^ 1996 or at discharge after first stay in hospital for schizophrenia. | At discharge after first stay in hospital for schizophrenia. | January 1^st^ 1995 or thereafter at the moment of first diagnosis. | After January 1^st^ 1995 at the moment of first diagnosis. |
| Observation period | January 1^st^ 1996 to December 31th 2006 | January 1^st^ 1996 to December 31th 2015 | January 1^st^ 1996 to December 31th 2015 | January 1^st^ 1995 to June 31th 2014 | January 1^st^ 1995 to June 31th 2014 |
| Years of observation | 11 | 20 | 20 | 19.5 | 19.5 |
| Duration of follow-up, in years | Mean: 8.6 | Mean not reported  Median: 14.1 | Mean not reported  Median: 11.1 | Mean: 11.3 | Mean: 8.8 |
| N | 66,881 | 62,250 | 8,719 | 50,881 | 22,110 |
| Patient-years | 573,860 | 765,190 | 80,703 | 572,617 | 195,461 |
| Deaths totaal | 19,735 | 13,889 | 1,160 | 13,387 | 3,612 |
| Deaths suicide | 637 | Not reported | Not reported | 1,050 | 479 |
| Deaths cardiovascular | 1561 | Not reported | Not reported | 3,601 | 917 |
| Standardized Mortality Rate | 34.4/1000 patient-years | 18.2/1000 patient-years | /1000 patient-years | 23.4/1000 patient-years | 18.5/1000 patient-years |
| Excluded deaths during hospital admission | After 2 days of admission: 7,358  (37.3%) | After 7 days of admission: numbers not reported | After 7 days of admission: numbers not reported | After 14 days of admission: 1,439 (10.7%) | After 14 days of admission: 375 (10.4%) |
| Standardized Mortality Rate for users of antipsychotics | 26.8  (11,458 deaths per 426,930 patient-years) | 14.3  (8,264 deaths per 577,417 patient-years) | 9.8  (540 deaths per 55,069 patient-years) |  | 20.6  (2549 deaths per 123,875 patient-years) |
| Standardized Mortality Rate for non-users of antipsychotics | 56.3  (8,277 deaths per 146,930 patient-years) | 30.0  (5,635 deaths per 187,773 patient-years) | 24.2  (620 deaths per 25,634 patient-years) |  | 14.9  (1,065 deaths per 71,586 patient-years) |
