# Supplementary material for: Clozapine and mortality: A comparison with other antipsychotics in a nationwide Danish cohort study
Source: Acta Psychiatr Scand. 2020 Dec 25;143(3):216–26. doi: 10.1111/acps.13267 (PMC7986383; doi:10.1111/acps.13267)
Supplement: Supplementary file 6 — Appendix S1 [file ACPS-143-216-s001.docx]

Appendix 1, extra information (in bold) on materials and methods.

**Outcomes**The primary outcome was all-cause mortality. Secondary outcomes were suicide **(ICD-10 codes X60-84 and Y10-34)** and cardiovascular mortality **(ICD-10 codes G45-46 and I10-99)**.

**Exposure**

Treatment was categorized as follows: 1) clozapine (reference); 2) olanzapine; 3) risperidone; 4) other Second-Generation Antipsychotics (SGAs: **sertindole, ziprasidone, lurasidone, quetiapine, asenapine, amisulpride, aripiprazole, paliperidone**); 5) First Generation Antipsychotics (FGAs: **chlorpromazine, levomepromazin, promazine, acepromazine, fluphenazine, perphenazine, prochlorperazine, periciazine, thiordazine, pipotiazine, haloperidol, melperone, pipamperone, bromperidol, flupentixol, chlorprothixene, tiotixene, zuclopenthixol, pimozide, penfluridol, loxapine, sulpiride**); 6) polypharmacy including clozapine (**polypharmacy is defined as prescriptions for 2 or more different antipsychotics in the same period**); 7) polypharmacy not including clozapine; 8) no antipsychotic medication; 9) hospital-delivered antipsychotic, type unknown: antipsychotics are distributed free of charge to patients sentenced to treatment and, since 2008, during the first 2 years subsequent to a diagnosis of schizophrenia. The type of antipsychotic is not known because the drug is not registered in the prescription registry. A small proportion of these patients will not be using any antipsychotic; 10) Drug Unknown: no data available because of hospitalization, inpatient drug use is not registered in the prescription registry. Episodes of drug use were censored on day 15 of hospitalization. We chose this time period because antipsychotic drugs are often continued at the start of a hospitalization and because their effects are likely to last during this period; 11) no use of antipsychotics.

**To define periods of antipsychotic use, we assumed that antipsychotic drug A was used from the moment of redemption for a duration of 90 days unless: a) a new prescription of the same drug was redeemed; b) another antipsychotic drug B was prescribed in the 90-days period without a subsequent or concurrently new prescription of drug A before the end of the 90 days period. A period of polypharmacy began when drug B was prescribed during the 90-days period of drug A, while drug A was prescribed again after the start of drug B. The use of one category of antipsychotic medication for a period of less than 3 months is one episode. If an antipsychotic drug is used for more than 3 months, each period of 3 months constitutes one episode. Episodes of drug use were censored on day 15 of a hospitalization, because during hospital stay no information on drug use is available. We took this arbitrary decision, because antipsychotic drugs are often continued during the first two weeks of a hospitalization and because their effects are likely to last during this period.**
We conducted separate analyses for current and cumulative use of antipsychotics. Both current and cumulative use are time-dependent variables and were recalculated at the time of each death event in the cohort, both for the patient who died and for those who were still alive at that time. The currently used antipsychotic was defined as the last drug that was prescribed before a death in the cohort, provided that death occurred after no more than 2 weeks of no use or no more than 2 weeks after hospital admission. Cumulative use was defined as a time-dependent variable as well and was recalculated at the time of each death in the cohort. For this measure, all episodes of use of a certain antipsychotic were aggregated and the total duration of these episodes was categorized as follows: 0-1, 1-3, 3-6, 6-10 years, and more than 10 years. Thus, one individual could contribute to several monotherapy or polypharmacy categories at different points in time during follow-up. However, when a death occurred, a subject was placed in only one category of cumulative antipsychotic use, namely, in the category of the drug that had been used the longest at that time. This implies that shorter periods of use of other antipsychotics at this point in time were disregarded. To illustrate this, after consecutively 2 years of olanzapine, 4 years of clozapine and 3 months of risperidone use, the patient is in the category “risperidone” in the analysis of current use and in the category “clozapine (3-6years)” in the cumulative use analysis. After 3 months of risperidone, 9 months of olanzapine and 6 months of clozapine use, a patient is in the “clozapine” category in the analysis of current use and in the category “olanzapine (0-1 year)” in the analysis of cumulative use. Hazard ratios were calculated with the category clozapine use as reference.

**Covariates**Baseline variables were age at start of follow-up, sex, primary psychiatric diagnosis **(ICD-10 codes F20, F25, F28 and F29, ICD-8 codes 295 and 299; the first diagnosis was used as the patient’s primary diagnosis unless it was later changed into an NAPD diagnosis ranking higher in the following hierarchy: 295/F20 was higher than F25, F25 was higher than 299, 299 was higher than F28 and F28 was higher than F29)**, and psychiatric hospitalization before follow-up (yes/no). We included the latter variable as a measure of the severity of illness. Duration of illness, i.e., duration since first registered diagnosis of NAPD at the time of cohort entry, was another baseline variable for members of the prevalence cohort. Time-dependent variables were substance use disorder **(ICD-8 codes 291, 303, 304; ICD-10 codes F10-F19)**, drugs for substance use disorder **(ATC codes N07B, excluding N07BA)**, mood disorder **(ICD-8 codes 296, 298.09; ICD-10 codes F30-F39)**, use of antidepressants **(ATC codes N06A)**, cardiovascular disorder **(ICD-8 codes 400-429, 432-438, ICD-10 codes G45-46, I10-99)**, drugs for cardiovascular disorders **(ATC codes B01, C01, C02, C03, C07, C08, C09 and C10)**, diabetes **(ICD-8 code 250, ICD-10 codes E10-14)**, drugs for diabetes **(ATC codes A10A, A10B)** and cancer **(ICD-8 codes 140-207, ICD-10 codes C00-C97)**. The time-dependent variables changed at the time of their first occurrence and were time-lasting (permanent). To illustrate this point, after a diagnosis of a mood disorder or the dispension of a drug for cardiovascular problems, this variable remained ‘yes’ for the rest of the follow-up period.

**Statistical analysis**

For the main analyses, we used an incidence cohort, because follow-up can be started at the moment of the first registration of a diagnosis of NAPD. The analyses for cumulative use were conducted in the incidence cohort only, because we did not have information on the use of antipsychotics before the start of follow-up in the prevalence cohort. Cox proportional hazards regression with time-dependent variables was used to estimate hazard ratios and 95% confidence intervals (CIs) for the association between exposure to antipsychotics and mortality (all-cause and cause-specific). All subjects were followed up from their first diagnosis, their 15th birthday, or from 1 January 1995, whichever occurred last, until death or 1 July 2014. To allow for the possibility of at least 1 year of follow-up, the latest entry date was 30 June 2013. **Right censoring was applied at death, emigration, other disappearance from the Danish centralized civil registration system, the 100 years birthday or the latest on July 1, 2014. Interval censoring was used for persons that emigrated and immigrated again within the study period.** Due to violation of the proportional hazards assumption, the Cox analyses were stratified by age at start of follow-up **(groups: 0-25, 25-35, 35-45, 45-55, 55-65, 65-75, 75+ years; the groups were merged as necessary)**, sex, Drug Unknown, and in the prevalence cohort also by (registered) duration of illness before the start of the follow-up **(0, 0-5, 5-10, 10-15, 15-20, and 20+ years)**. The proportional hazards assumption for the Cox regression models was tested and evaluated by graphical assessment of smoothed hazard estimates plots. Clozapine monotherapy was used as reference. The analyses were performed with Stata. A two-tailed p-value of <0.05 was considered statistically significant for all tests. Both the analyses of current use and the analyses of cumulative use were conducted for three types of mortality: (1) all-cause mortality; (2) mortality due to suicide; and (3) cardiovascular mortality.

We used two different types of adjustment in order to test the hypothesis that somatic comorbidity and the treatment thereof may influence the association between clozapine and mortality. In the first model, the results were adjusted for the time-fixed variables age at entry, sex, type of NAPD, and psychiatric hospitalization before start of follow-up, and for the time-dependent variables mood disorder, substance use disorder, malignant neoplasms, drugs for mood disorder, and drugs for substance use disorder. We adjusted for type of NAPD and psychiatric hospitalization, because they are proxies for illness severity. We adjusted for malignant neoplasms, in order to make sure that any difference between antipsychotics was not due to the occurrence of neoplasms. In the prevalence cohort, we also adjusted for time since first (registered) NAPD diagnosis. In the second model, the results were also adjusted for cardiovascular and diabetic comorbidity (diagnosis and dispension of drugs) as time-dependent variables.

Figure S1 Adjusted hazard ratios for mortality due to suicide after cumulative use of antipsychotics compared to cumulative use of clozapine (reference), between January 1995 and July 2014, in a Danish incidence cohort of patients with a non-affective psychotic disorder. Comparisons with clozapine were done within each of the groups distinguished by the same length of antipsychotic use: 0-1, 1-3, 3-6, 6-10 years, and more than 10 years. The observation period was from January 1995 to July 2014.

Adjusted for age, sex, type of non-affective psychotic disorder, mood disorder, substance use disorder, psychiatric hospitalization, somatic comorbidity and the treatment of somatic disorders.

Figure S2 Adjusted hazard ratios for cardiovascular mortality after cumulative use of antipsychotics compared to cumulative use of clozapine (reference), between January 1995 and July 2014, in a Danish incidence cohort of patients with a non-affective psychotic disorder. Comparisons with clozapine were done within each of the groups distinguished by the same length of antipsychotic use: 0-1, 1-3, 3-6, 6-10 years, and more than 10 years. The observation period was from January 1995 to July 2014.

Adjusted for age, sex, type of non-affective psychotic disorder, mood disorder, substance use disorder, psychiatric hospitalization, somatic comorbidity and the treatment of somatic disorders.
